# Cancer and the kidney: dangereoux liasons or price paid for the progress in medicine?

**DOI:** 10.18632/oncotarget.18094

**Published:** 2017-05-23

**Authors:** Jolanta Małyszko, Leszek Kozlowski, Klaudia Kozłowska, Maciej Małyszko, Jacek Małyszko

**Affiliations:** ^1^ Second Department of Nephrology and Hypertension with Dialysis Unit, Medical University of Bialystok, Bialystok, Poland; ^2^ Department of Oncological Surgery, Ministry of Interior Affairs, Bialystok, Poland; ^3^ First Department of Nephrology and Transplantology with Dialysis Unit, Medical University of Bialystok, Bialystok, Poland

**Keywords:** chronic kidney disease, malignancy, acute kidney injury, chemotherapy, kidney function

## Abstract

A long time ago, the links between renal disease and malignancy were observed, however, quite recently, their importance was recognized and ‘new’ subspecialty in nephrology, namely ‘onconephrology’ was established. In the XXI century, patients with malignancy make up the most growing number of the subjects seen for nephrology consult and/or critical care nephrology services. A plethora of renal problems may be found in patients with malignancy. They may influence not only their short-term outcomes but also the adequate therapy of the underlying oncological problem. Thus, all these kidney-related issues pose an important challenge for both specialities: oncology and nephrology. In the review a spectrum of acute and chronic renal injury caused by the malignancy is presented as well as the associations between renal disease and cancer. Assessment of kidney function and its importance in patients with malignancy is also discussed as medical oncologists should check the appropriate dose of chemotherapeutic drugs in relation to the actual renal function before prescribing them to the patients. Moreover, effects of kidney function on outcomes in oncology is presented. In addition, nephrology services should better understand both the biology of malignancy with its treatment to become a valuable part treating team to yield the best possible outcome. It is important for nephrology services to be acknowledged and to take an active participation in care of oncology patients.

## INTRODUCTION

In the XXI century patients with malignancy make up the most growing number of the subjects seen for nephrology consult and/or critical care nephrology services. The outstanding progress in the therapy of malignancy presents new possibilities and challenges for both nephrologists and medical oncologists. It is important for nephrology services to be acknowledged and to take an active participation in care of oncology patients. In addition, nephrology services should better understand both the biology of malignancy with its treatment to become a valuable part treating team to yield the best possible outcome.

A long time ago, the links between renal disease and malignancy were observed, however, quite recently their importance was recognized and ‘new’ subspecialty in nephrology, namely ‘onconephrology’ was established [1]. Chronic kidney disease-CKD is often diagnosed in the general population [2], however, the its incidence and prevalence among patients with malignancy was not extensively studied and data were limited. Half of the century ago, increased incidence of cancer in CKD patients has been discussed by Sutherland et al [3]. Other reports from the last century have also linked chronic kidney disease with an increased incidence of cancer [4–10]. In XXI century, Cengiz [11] reported that in the last 20 years, prevalence of solid tumors was 6.7% in the population of 2817 subjects with CKD, including 199 subjects on hemodialyses. It is of interest that 71% of the hemodialyzed patients were diagnosed with tumors in the first year of the therapy, while in 84% of patients with CKD, tumors were detected in less than 10 years after diagnosis of CKD. The most common were urologic malignancy followed by parathyroid adenoma and skin cancer in this population studied.

**Figure 1:**
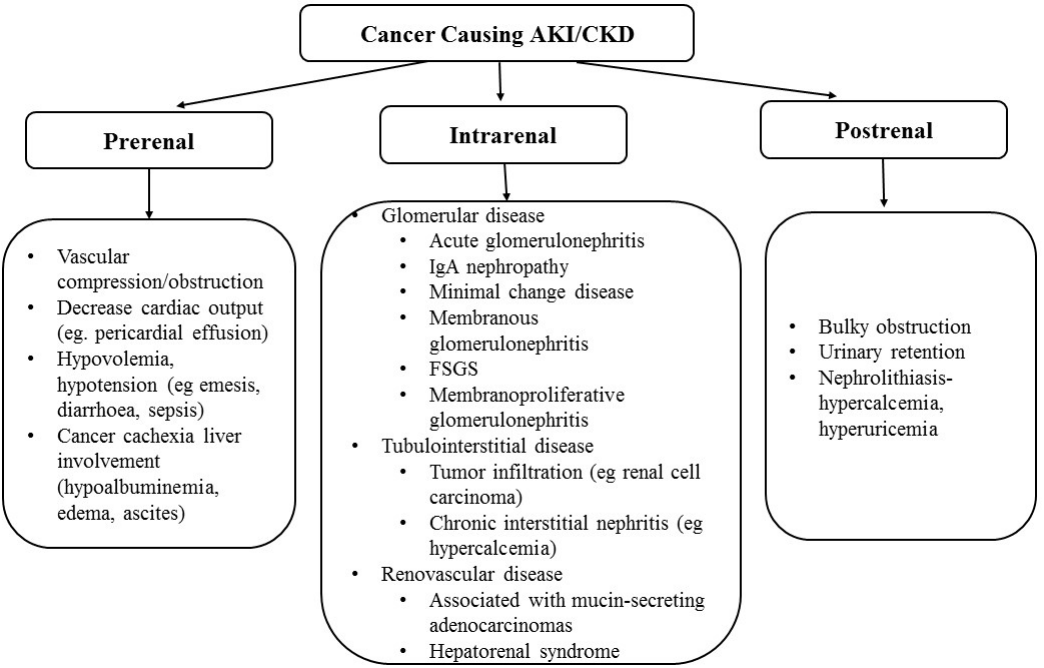
Cancer causing acute kidney injury (AKI) and/or chronic kidney disease (CKD)

**Figure 2:**
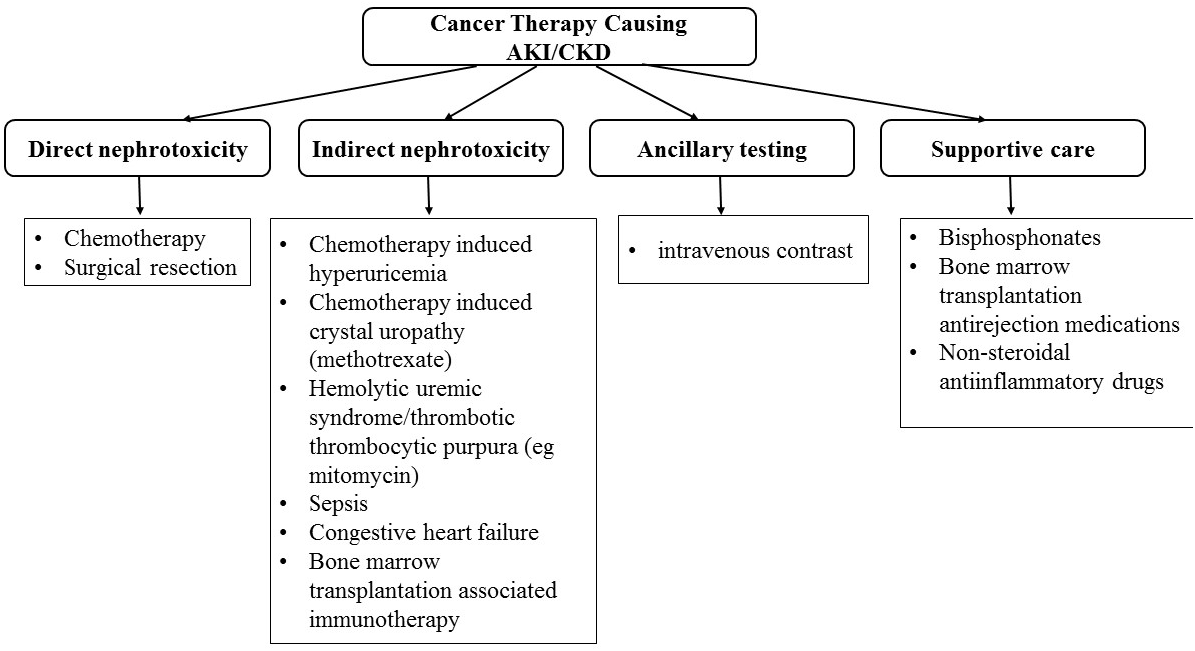
Cancer therapy causing acute kidney injury (AKI) and/or chronic kidney disease (CKD)

A plethora of renal problems may be found in patients with malignancy. They may influence not only their short-term outcomes but also the adequate therapy of the underlying oncological problem. Thus, all these kidney-related issues pose an important challenge for both specialities: oncology and nephrology. Indeed, the incidence rates for many malignancies are increased and amelioration in cancer mortality due to more effective chemotherapy, including targeted drugs, and treatment with stem cells, caused in a rise in population of cancer survivors [12]. Some of these survivors develop acute kidney injury-AKI or CKD due to either cancer (Figure 1) and/or its therapy (Figure 2) [13]. The kidneys may be thus directly or indirectly damaged by the malignancy or one or more of the novel therapeutics that prolong lives, however at the cost of developing AKI or CKD. In addition, multiorgan failure may be also seen in cancer patients. In consequence, they may require intensive care unit-ICU care and renal replacement therapy-RRT. In the setting of advanced malignancy complicated by multiorgan illness, appropriateness of aggressive treatment in ‘‘futile situations’’ and the role of palliative therapy remains the open question. Thus, care for oncology patients has become more specialized and complicated, requiring collaboration among nephrology, medical oncology, intensive therapy, and palliative care. Then the question of persistent therapy and end-of life care appears as well as the continuation of RRT in advanced malignancy.

### Acute kidney injury in patients with malignancy

AKI and disturbances in electrolyte are the most common feature of kidney disease that are found in a patient with malignancy in a hospital setting. AKI in this population is linked with high morbidity and mortality. AKI incidence in these vulnerable patients depends upon type of malignancy (solid tumor or hematological malignancy), severity of underlying disease, complications of the disease and therapy. Several factors may potentiate the risk of AKI in these patients such as dehydration due to vomiting, diarrhea, obstruction of urinary tract, fluid and electrolyte disturbances, contrast agent administration, nonsteroidal antiinflammatory drugs (NSAIDs), nephrotoxic antibiotics, renal toxicity of some chemotherapeutic and targeted drugs [14–17]. Moreover, critical illness in cancer patients is linked to the increased incidence of AKI and need for RRT relative to the population with similar severity of illness but without cancer [18–20]. AKI is this population is mainly due to therapy or malignancy itself [21]. AKI combined with critical illness in cancer patients yield worse outcomes, with mortality rates up to 85% in cases requiring renal replacement therapy [20]. Salahudeen et al. [22] studied 3558 subjects admitted to the Anderson Cancer Centre (Texas, USA) in a period of 3 months in 2006. They found that 12% were diagnosed with AKI. In the multivariate analysis, diabetes, chemotherapy, intravenous contrast administration, hyponatremia and use of antibiotic(s) were found to predict AKI development. Complications, comorbidities or exposures present in patients with malignancy before hospitalization posed higher risk of AKI than chemotherapy. Canet et al. [23] reported that AKI incidence in subjects with high-grade hematological malignancy was as high as 68.5% using RIFLE criteria (risk, injury, failure, loss of function, end-stage kidney disease-ESRD) with hypoperfusion, acute tubular necrosis, tumor lysis syndrome, nephrotoxins and hemophagocytic lymphohistiocytosis accounting for 91.5% of cases. Similarly to other AKI patients, impairment in kidney function previously considered trivial, has an equal prediction value of a unfavorable outcome in critically ill subjects with cancer [7]. Even a small increase in creatinine up to 10% (0.2 mg/dL–17.6 μmol/L) was linked to extended ICU stay and higher death rates. Subjects with an elevated rise in serum creatinine (SCr), more than 25% within the first 72 hours of ICU admission had a 2-fold increase in mortality during hospitalization (14.3% vs 30.1%, P < 0.001). This unfavorable outcome in patients with increased SCr is not solely due to the severity of disease or presence of other risk factors. It also should be taken into account, that the other comorbidities found in the patients with malignancy, may have a direct effect the on care of oncological subjects, type of the therapy and its efficacy. They also are predictive in patient outcomes, due to their possible interactions with the malignancy and developing frequently a more morbid condition than that caused by the malignancy alone [8–10]. Data on AKI epidemiology in oncological patients are limited, however, it appears that the incidence of AKI is at least 3-fold higher in this population relative to that without cancer [6, 7, 14, 15]. AKI in patients with malignancy may due either to ordinary causes, or there are unique or more common causes of AKI such as lymphomatous infiltration of the kidneys, cast nephropathy in multiple myeloma and monoclonal gammopathies, tumor lysis syndrome, mainly in malignancies with high tumor burden and rapid cell turnover [1, 17]. In addition, in patients treated with hematopoietic cell transplantation there are several unique causes of AKI [1, 17]. Outcomes in cancer patients with AKI depend upon several factors. Soares et al. [24] reported that ICU, hospital, and 6-month mortalities in patients with malignancy and AKI admitted to the ICU were 55%, 64%, and 73%, respectively. In this study, 6-month mortality was associated with an age of 60 years or older (HR 1.36; 95% CI 1.00-1.84), an Eastern Cooperative Oncology Group- ECOG status of 2 to 4 (HR 1.66; 95% CI 1.22-2.26), more than 1 organ dysfunction (> organs, HR 4.07; 95% CI 1.94-8.54), uncontrolled cancer (HR 1.61; 95% CI 1.10-2.11), and severity of kidney failure (HR 1.77; 95% CI 1.26-2.49) [24].

Kidneys clear same chemotherapeutics and AKI affect pharmacokinetics of these drugs. It may lead to their toxicity. On the other hand, RRT may lead to subtherapeutic levels of chemotherapeutics making treatment potentially ineffective. In addition, AKI may alter the levels of antibiotics, narcotics and other ancillary drugs leading to potential adverse effects. Thus, kidney function should be monitored carefully and drugs dose adjusted appropriately. In cases when not nephrotoxic drugs are available, it should be the preferred therapeutic option.

### Prerenal AKI

AKI due to prerenal causes is a common finding in patients with malignancy [7]. In this population, AKI may be due to true dehydration, resulting from vomiting, diarrhea or sepsis. AKI due to dehydration related to malnutrition associated with antineoplastic therapy is commonly observed [7]. In sepsis, impaired perfusion and prerenal AKI may be due to hypotension and vasodilation due to either sepsis or administration of vasoconstrictory drugs, i.e. norepinephrine or vasopressin. In addition, prerenal AKI may be also caused by drugs such as diuretics, angiotensin II receptor blockers, angiotensin-converting enzyme inhibitors or NSAIDs used for either the cancer or other situations. Thus, physicians should be aware of the risks and benefits of continuation of these medications in oncological subjects. Prevention of the prerenal AKI is adequate hydration and avoidance or withdrawal of potentially nephrotoxic agents.

### Postrenal AKI

Intratubular or extrarenal obstruction are frequent causes of AKI in patients with cancer [17, 25]. Conversely, malignancy should be considered in any patient not known to have cancer who presents with bilateral urinary tract obstruction that is not associated with urolithiasis. Obstruction could be either intratubular or extrarenal. Intratubular obstruction can be caused by uric acid crystals (in tumor lysis syndrome), light chain casts, or crystallization of certain drugs i.e high dose methotrexate. Obstruction of the bladder outlet or urether(s) is more frequent in malignancies relative to the general population [26]. Extrarenal obstruction can be caused by a wide range of malignancies i.e. bladdder, prostate, uterus and cervix cancers may cause obstruction of the urinary tract and postrenal AKI, and usually indicates metastatic disease [17, 25]. Ureteral obstruction due to retroperitoneal fibrosis can be also secondary to malignancy. Patients with cancer may also develop urinary tract obstruction that is unrelated to the malignancy (eg, benign prostatic hypertrophy in men). The most common clinical presentation is anuria, flank pain, a palpable mass or palpable bladder. Urinary sediment is usually bland. In a case of partial obstruction, anuria may not be present. However, hyperkalemia with nonanion gap metabolic acidosis may suggest renal tubular acidosis due to obstruction [27]. On sonography, hydronephrosis or hydroureter are most common findings. However, in a case of obstructive AKI due to retroperitoneal fibrosis, malignancy or its treatment, hydronephrosis or hydroureter may not be present. It should be also stressed that radiotherapy of the pelvis or abdomen may also lead to retroperitoneal fibrosis. Percutaneous nephrostomy or stenting is performed to relief obstruction of the urinary tract, however, recovery is influenced by the severity and duration of the obstructive AKI.

### Renal AKI

Glomerular, tubulointerstitial, and vascular diseases may cause renal function impairment in patients with malignancy. In differential diagnosis type of malignancy and type of chemotherapeutic agents are to be considered. The most common glomerular pathology in malignancy include vasculitis associated with antineutrophil cytoplasmic antibodies (ANCA), membrano-proliferative or membranous glomerulonephritis and thrombotic microangiopathy (TMA) [17, 25]. Acute tubular necrosis due to ischemia may be caused by severe dehydration, hypotension or sepsis leading to prerenal AKI and then due to the severity and duration to intrinsic AKI. Chemotherapy-induced kidney injury are presented in the review elsewhere [28].

### Novel anti-cancer agents-related kidney problems

It has been recognized that novel treatment with targeted drugs offer superior patient survival rates compared with standard chemotherapy. The most commonly used cancer therapies are targeted to proteasome, vascular endothelial growth factor-VEGR and its receptor- -VEGFR, epidermal growth factor receptor- EGFR, human epidermal growth factor receptor-2-HER2, dimerizations of HER2, v-Raf murine sarcoma viral oncogene homolog B- BRAF, anaplastic lymphoma kinase-ALK, programmed cell death protein 1-PD-1 and its ligand PDL-1, receptor activator of nuclear factor kappa beta ligand- RANKL, and mammalian target of rapamycin-mTOR. However, the downside of the targeted therapies and immunotherapy is unique adverse renal toxicity, well different from that of the conventional chemotherapy [29–31]. This toxicity was attributed to co-expression of same target molecules by both normal and cancer cells. VEGF pathway inhibitors include VEGF ligand inhibitors, which bind to and inhibit ligand binding to the VEGFR, thus preventing activation of the receptor as bevacizumab or targeted to VEGFR2 ramucirumab; and antiangiogenic small molecule tyrosine kinase inhibitors-TKIs (sunitinib, sorafenib, pazopanib, ponatinib, axitinib, cabozantinib, lenvatinib, vandetanib), which block the intracellular domain of the VEGFR and a soluble recombinant decoy that binds to circulating VEGF; aflibercept (VEGF-Trap) [32]. The major renal adverse effect of this class is proteinuria, even nephrotic syndrome with hypertension as reviewed elsewhere [33]. In patients with proteinuria receiving VEGF-targeted agents, TMA, collapsing glomerulopathy, proliferative glomerulonephritis and isolated reports of cryoglobulinemic and immune complex glomerulonephritis were diagnosed on the kidney biopsy [34–36]. Antiangiogenic TKIs may cause also proteinuria, TMA, acute and chronic interstitial nephritis [33, 37]. Monoclonal antibodies targeting the EGFR (cetuximab, panitumumab, necitumumab, matuzumab) are associated with the progressive development of hypomagnesemia due to renal magnesium wasting [38–40]. Immune checkpoint inhibitors (ICI) represent major improvements in patient outcomes in oncology. Cytotoxic T-lymphocyte antigen 4 -CTLA-4 and PD-1 are two essential immune checkpoint receptors. Ipilimumab and tremelimumab (anti-CTLA-4-blocking antibodies) and pembrolizumab and nivolumab (antibodies targeting PD-1 receptors) have already been approved in several malignancies. Proteinuria, hypertension, renal failure and acute interstitial nephritis were reported in subjects given anti-PD-1 antibodies [41–44]. In patients treated with CTLA-4 antibodies, nephrotic syndrome, acute tubular injury, acute tubular necrosis, acute interstitial nephritis and AKI have been reported [45–50]. The observed acute renal damage can be reversed upon drug discontinuation and introduction of systemic steroid therapy.

### Use of bisphosphonates in cancer-related bone disease

Bisphosphonates are used to prevent bone resorption. Pamidronate and zoledronate are approved for bone events prevention in patients with advanced malignancy. Nephrotoxicity of bisphosphonates include mainly nephrotic syndrome, glomerulopathies such as collapsing focal segmental glomerulosclerosis (FSGS), FSGS-non specified and minimal change disease acute tubular necrosis and acute kidney injury [51–55].

### Contrast-induced nephropathy

Contrast-induced nephropathy (CIN) is an important drawback following administration of intravascular iodinated contrast agent [56]. In the majority of studies, the definition of CIN is as an absolute (≥0.5 mg/dL) or relative (≥25%) rise in SCr within 48–72 h after administration of iodinated contrast agent, when the rise in SCr could not be explained otherwise [56]. Patients with cancer are treated with variety of nephrotoxic medications (chemotherapeutics, targeted drugs, antibiotics, analgesics and others). Additionally, other problems like anemia, hypercalcemia and hyperuricemia may also contribute to development of kidney damage in patients with malignancy. Computed tomography with contrast (CT) appears to be standard and most common imaging procedure in oncology to monitor and evaluate the therapeutic response. Thus, the risk of CIN in patients with malignancy could be increased. Many cytotoxic and targeted medications as well as drugs for supportive care are contraindicated in the presence of impaired kidney function. Worsening of kidney function precludes or delays appropriate antineoplastic therapy. Cytotoxic drugs may be responsible for acute endothelial damage rather than toxic effects on kidney. Their administration can result in vasoconstriction, arterial hypertension and tissue ischemia as they effect the vasomotor activity of vascular smooth muscle via the nitric oxide synthesis, and adenosine and endothelin release. In turn, oxygen free radicals and lipid peroxidation may follow leading to inflammation, endothelial injury and thromboembolism [57–63]. Cytotoxic agents may affect endothelial and vascular structures leading to ischemic heart disease, cerebrovascular disease, venoocclusive syndrome, Raynaud’s phenomenon and capillary leakage syndrome [64]. Bevacizumab was reported to influence significantly renal endothelium and vasculature [64]. There are several mechanisms of CIN development by contrast agents. They lower the glomerular filtration rate (GFR) and renal medullary blood flow as they exhibit vasoconstrictory effects on kidney vasculature [56–60]. Moreover, they are responsible for tissue ischemia/hypoxia due to increased adenosine and endothelin concentrations [65]. Inflammation due to renal tissue injury and free oxygen radical synthesis may also lead to CIN after administration of contrast agents [66, 67]. A few studies evaluated CIN prevalence in cancer patients. Cheruvu et al. [68], using the retrospective data of subjects with malignancy, reported CIN prevalence in 9% of the subjects with preexisting kidney disease (irreversible CIN was observed in half of these patients) and in approximately 5% of those without kidney disease. It should be stressed, that in their institution, a National Cancer Institute, they used iodixanol, an iso-osmolar, dimeric, non-ionic contrast agent in CKD patients [68]. In this population, many additional risk factors for CIN development were present and related to the underlying disease (ie, multiple myeloma and nephrotoxic chemotherapeutics) [68]. Cicin et al. [69] reported the 4.5-fold higher risk of CIN in subjects undergoing CT within 45 days after the course of chemotherapy relative to those not given chemotherapy or undergoing CT more than 45 days after the course of chemotherapy. They used low-osmolar, non-ionic contrast agents i.e. iopromide (428 mOsmol/l) or iohexol (465 mOsmol/l). They suggested that patients undergoing CT with contrast within 45 days after chemotherapy were at the increased risk for CIN. Among chemotherapeutics, only the combination of bevacizumab/irinotecan appeared to bear the increased risk for CIN. Ng et al. [70] assessed the admissions to an oncology ICU of 3,848 subjects and compared CT with and without contrast medium with no CT. They found that SCr increased in 55% of patients after CT (with and without contrast agent) [70]. No significant difference between the two groups (with and without contrast agent) was detected as a probable reflection of the critical illness in the studied population. The prevalence of CIN was approximately 16–17%. Absolute change in SCr between matched CT groups (with and without contrast agent) did not differ significantly. The authors concluded that IV contrast agent administered to patients with relatively normal kidney function in oncologic ICU caused a rise in SCr. However, the increase in SCr was not beyond that of simply performing CT or of a matched non-CT group in ICU. Crucial studies were published by McDonald et al. [71–73] who questioned the concept of CIN after contrast media exposure. Over than 100000 patients, including cancer population were retrospectively analyzed in this multiple propensity score matched trials, each trial enrolled over 10000 patients. In the large, retrospective, single-center study, they observed equal risk of AKI among patients receiving contrast enhanced computed tomography if compared to unenhanced computed tomography. Systemic review published in 2013, based on studies concerning AKI incidence among patients underwent enhanced and unenhanced CT, showed similar risk of AKI in both group of patients [71]. Even the risk of dialysis and mortality was comparable in both groups [71]. Moos et al. [74] in the meta-analysis reported a low incidence of CIN after CT. Development of CIN was predicted by the presence of kidney failure, diabetes, cancer, old age and therapy with NSAIDs. They evaluated 42 publications with 18,790 subjects (mean age 61.5 years, ranged 38–83 years), with mean baseline eGFR of 59.8 mL/min/1.73m^2^ (range: 4 - 256 mL/min/1.73m^2^). In 45.0% of patients eGFR was less than 60 mL/min/1.73m^2^, 55.2% were hypertensive; 20.2% were diabetic (DM) and 6.5% suffered from congestive heart failure (CHF). They stressed that although many risk factors are mentioned in the guidelines, only a few predicted CIN development after IV iodinated contrast agent administration for CT, namely: kidney failure, DM, age > 65 years and NSAIDs. Additionally, despite significant association between CIN development and cancer was revealed, the present guidelines did not contain such an information. The value gained and the risk incurred by imaging studies in general, and radiocontrast-enhanced imaging studies in particular, remain critically important questions in many medical and surgical subspecialties including oncology. Although data in oncology are very limited, it is suspected that, on the basis of existing assumptions regarding attributable risk, diagnostic studies and some interventions that might save or improve lives are being withheld from patients owing to an exaggerated fear of radiocontrast nephropathy. Therefore, the benefit of a contrast-enhanced study with the risk, likely low but likely not zero, of radiocontrast administration on the kidney should be carefully estimated as to date there have been no randomized studies of the risk of radiocontrast administration in oncology field.

### CKD in patients with malignancy

#### Prevalence of CKD in patients with malignancy

In the last years, a remarkable number of targeted drugs have shown their efficacy and benefits in various malignancies together with improved outcomes such as progression-free and/or overall survivals. Prevalence CKD is reported to be high in patients with malignancy [75], but the renal effect of new targeted therapies have not been not widely studied. CKD prevalence of ∼33 and 27%, respectively, was reported by Dogan et al. [76] and Launay -Vacher et al. [77]. IRMA-1 (Insuffisance Rénale et Médicaments Anticancéreux; Renal Insufficiency and Anticancer Medications) study included 4684 subjects with malignancy. In this study, 50–60% of the subjects had an abnormal renal function (GFR<90 ml/ min/1.73 m^2^), whereas SCr was normal in most patients [50]. These results emphasize the CKD incidence is high in subjects with malignancy. In France, in the ‘IRMA’ studies, a prevalence of a GFR below 90 ml/ min/1.73m^2^ was 52.9% in IRMA-1 [78] and 50.2% in IRMA-2 [79], in a cohort of 5000 subjects with different types of malignancy. According to KDIGO (Kidney Disease Improving Global Outcomes) definition [80], the prevalence of stage 3 to 5 CKD, excluding RRT, was also high reaching 12.0% in IRMA-1 and 11.8% in IRMA-2, respectively [78, 79]. Huang et al. [81] reported that 87% patients with renal cancer, had eGFR <90 ml/min/1.73m^2^. The study was performed in a cohort of 662 subjects with a renal cortical tumor subjected to either partial or radical nephrectomy. In addition, eGFR <60 ml/min/1.73m^2^ was reported in 26% [81]. Prevalence of CKD ranged from 16.1% to 25.0% in patients with malignancy in Belgium [82], United States [83], and Japan [84]. In the IRMA-1 study, CKD was highly prevalent reaching approximately 50% in either breast, colorectal, lung, ovarian, or prostate cancers [85–87]. The Belgian Renal Insufficiency and Anticancer Medications (BIRMA)[82] study was a large, national, multicenter and retrospective trial. It was performed to evaluate CKD prevalence in Belgian patients with malignancy [82]. In addition to IRMA-1 study, the rationale for BIRMA trial was to describe the type and dosage of the antineoplastic therapy prescribed according to kidney function and to evaluate the interactions between kidney function and anemia, previous anticancer therapy, history of kidney disease, and metastases [82]. The study was performed on 1218 patients. Elevated SCr (≥1.2 mg/dL) was found in 14.9% of patients, but in 64.0% of them eGFR was below 90 ml/min/1.73m^2^. In all, 78.6% of patients (*n*=1087) were administered at least one drug subjected to dose adjustment and 78.1% received at least one drug known to be nephrotoxic. This is of interest, that 56.5% of patients with CKD treated with chemotherapeutics requiring dose adjustment in case of CKD, had no dose reduction [82]. From this study it appears, that prevalence of CKD is high in patients with malignancy and is routinely underestimated by clinicians as they assess kidney function using serum creatinine level only. Moreover, 80% of the patients were administered drugs with potential nephrotoxicity and/or for which dose had to be adjusted in CKD. Therefore, the dose of chemotherapeutics should be checked in advance by medical oncologists taking into account the actual kidney function. In the recent study, Yang and et al. [88] demonstrated that 32.4% of patients with newly diagnosed cancer exhibited chronic kidney disease. In addition, renal function was inversely related to all-cause mortality. Moreover, eGFR below 60 mL/min/1.73m^2^ was an independent predictor of mortality relative to eGFR ≥60 mL/min/1.73 m^2^, and it was dependent upon cancer site. After adjustment for confounders, eGFR<60 mL/min/1.73m^2^ was associated with higher mortality risk among patients with hematologic malignancy and gynecological cancer. They stressed that they enrolled incident rather than prevalent patients and eGFR was obtained at the time of diagnosis, to rule out the effect of anticancer treatment on kidney function.

#### Associations between cancer and CKD

Wong et al. [89] studied a cohort of 3654 subjects and assessed the relation between eGFR and risk of cancer. They found that in men, but not in women, with eGFR lower than 55 ml/min/1.73m^2^, a risk for cancer was significantly higher [89]. In particular, lung and urinary tract cancer risk raised by 29% for each 10 mL fall in eGFR (estimated by Modification of Diet in Renal Diseases-MDRD formula). Danish registry study assessed the risk for cancer over two 8-year periods of time: 1993–2000 and 2001–2008. The authors found that in the studied periods, the incidence of malignancy per year of risk did not increase significantly, 3.1% versus 2.6%. However, the prevalence of cancer rose gradually by 35% from 10.4% in the earlier period to 14.0% in the later period [90]. The most common malignancies in this study were skin cancers (basal cell and squamous-cell), breast cancer, cervical cancer, melanoma, followed by colon, respiratory tract, bladder, prostate, and kidney cancers [91]. On the basis of these findings, it appears that CKD itself is a risk for cancer, dialyses or kidney transplantation, as reported previously [92, 93]. In breast, colorectal, lung, ovarian, and skin cancers, prevalence of CKD was increased [91–93] In addition, breast, cervix, colon, and kidney are more common in CKD than in the general population [91–93]. It is of importance that for these malignancies targeted drugs became available and introduced into the clinical practice. Therefore, the issue of renal safety is very important.

#### Cancer and renal replacement therapy

Patients on RRT are dying mainly due to o cardiovascular disease and infections, while malignancy is relatively common in this population. About 6% of the incident hemodialyzed subjects in the USA have malignancy as a comorbidity [94]. Despite the population of patients requiring RRT is growing and ESRD is associated with an increased risk of malignancy, data on the on the optimal management of ESRD patients with cancer is limited. Butler et al. [95] using US adult patients enrolled in Medicare's ESRD program hemodialyzed within the period from April 1, 1995, through December 31, 2010, assessed 5-year cumulative cancer incidence since RRT. They reported that 5-year cumulative incidence of any malignancy was 9.48% and was elevated for certain subgroups: elderly, non-Caucasians non-Latino, males, nondiabetics, recent hemodialysis therapy, and history of kidney transplant evaluation. They also suggested a high burden of malignancy in the hemodialyzed population compared to the US general population, as well as with the 4.4% estimate among US transplant recipients as reported by Hall et al [96]. Lin et al. [97] used the data from the Taiwan National Health Insurance Research Database on subjects who initially received RRT between January 1997 and December 2004. They showed that the RRT group revealed a significantly higher 7-year cancer incidence rate than did the general population and risk for blood, liver, colorectal, oral, breast, renal, upper urinary tract, and bladder cancer development was significantly higher than in the general population. In Europe, Bechade et al. [98] used the data from cancer registries and hospital databases in one French region and searched for subjects with an incident malignancy between 2001 and 2008 who started RRT. They found that the incidence rate of RRT in the population of incident malignancy was 370 per million population/year (74 events/199,809 person-years). Age-adjustment standardized incidence ratio was 1.26, (95 % CI 0.98-1.57, p = 0.55). In contrast to the American [95] and Taiwan [97] counterparts, they suggested that the standardized incidence ratio of chronic dialysis initiation was not significantly differed between patients with malignancy and the general population [98]. There are several explanations for increased cancer incidence in the dialysis population such as ESRD-associated immunodeficiency and nutritional abnormalities [99–109]. Interactions between immune dysfunctions due to of uremia and ESRD with established risk factors such as UV radiation, tobacco, or alcohol may also contribute to the excess cancer risk in CKD [100, 103]. Recently, there has been a focus on the potential role of erythropoietin-stimulating agents, commonly used to manage anemia; in carcinogenesis, they are known to activate erythropoietin receptors on the surface of cancer cells. Additionally, erythropoietin-induced angiogenesis may promote tumor growth [110, 111]. Nephrologists who care for ESRD population face the challenges including delays in diagnosis of malignancy, unclear utility of malignancy screening, and dilemmas in diagnostic imaging [112]. In addition, in a case of advanced or refractory malignancy, both nephrology and oncology specialists may cope with the issue of ethically complex palliative care as well as withholding of RRT [113–117]. There are several mechanisms to explain the higher increased incidence of malignancy in ESRD population. There are several confounding factors which may affect the diagnosis and evaluation of cancer in ESRD such as delayed symptomatic presentation, a non-validated and unclear utility of tumor markers, dilemmas in imaging studies, and lack of prognostic data. Several important cancer presentations may be omitted in ESRD such as hypercalcemia, hyperphosphatemia, pruritus, anemia with severity inadequate to the kidney function and oliguria/anuria [118]. Serum cancer antigen 125 (CA-125) may be a useful tumor marker however, it can be elevated in ascites, even in non-malignant settings [119]. Although prostate-specific antigen (PSA) is not removed by dialysis, fluctuations and rises in PSA levels could be observed secondary to hemoconcentration or disturbances in binding proteins following RRT [120]. Imaging studies requiring contrast are commonly performed for diagnosis, staging, or monitoring of malignancy. As with iodinated contrast agents, the European Society of Urological Radiology (ESUR) does not recommend any specific timing for HD following nuclear magnetic resonance imaging with gadolinium.

There is uncertainty on validity of conventional prognostic factors and outcome data for specific malignancy in ESRD population on RRT due to the absence of randomized clinical trials. Several aspects of antineoplastic regimen in RRT are important to consider such as selection of chemotherapeutic agent, dose adjustment, timing of dialysis in relation to administration of chemotherapeutic agent, vascular access for RRT and chemotherapeutic regimen, type of dialysis and staff safety considerations. The kidney clearance of a cytotoxic drugs is important in dialyzed patients. RRT can affect clearance of chemotherapeutics in several ways than just a simple first-order kinetics. Cytotoxic agent could be cleared by dialysis. It may affect the dose given as well as the timing and characteristics of the subsequent dialysis. The preservation of vascular access for RRT is crucial and especially demanding in a patient receiving cytotoxic drugs, but the effects of quickly administered chemotherapeutics through an arterialized vein have not been adequately assessed. Thus insertion of the port for chemotherapy may be worth to consider, however, possible complications of having both port and central venous catheter, in particularly on the same site should be taken into account. Data on chemotherapeutic drugs in ESRD are very scarce. For some cytotoxic medications, it appears that ESRD subjects can tolerate standard treatment. It seems very important that a systematic approach to investigate anticancer treatment in the growing RRT population is of utmost importance, particularly for the frequent malignancies such as lung, breast, colorectal, and prostate cancers for which the data are in general under-represented relative to other less common cancers such as transitional cell cancer, cancer of the testis and leukemia.

#### Assessment of kidney function in patients with malignancy

Assessment of glomerular filtration rate is complex, cumbersome and time consuming to perform on everyday basis. However, SCr should be used to estimate GFR in subjects with stable kidney function [121]. Moreover, precise GFR is not required for most clinical settings and it is unrealistic. However, in some clinical situations it appears to be reasonable to consider measuring GFR i.e dose adjustment of medications, especially toxic medications with narrow therapeutic indices, such as chemotherapy. Inulin is the gold standard of exogenous filtration marker, however, using alternative filtration markers (such as radioactive or nonradioactive; iohexol, iothalamate, ethylenediaminetetraacetic acid- EDTA or diethylenetriaminepentaacetate-DTPA), bolus administration of the marker (intravenous or subcutaneous), spontaneous bladder emptying and plasma clearance GFR could be estimated simpler and less cumbersome than inulin clearance [80]. At present, the most common methods utilized are the creatinine clearance and estimation formulas based upon SCr such as the Cockcroft-Gault formula, the Modification of Diet in Renal Disease (MDRD) study formula, and the Chronic Kidney Disease Epidemiology Collaboration (CKD-EPI) formula [80]. Both the creatinine clearance assessment and estimation formulas rely upon SCr as a marker of kidney function. Unfortunately, SCr is an unreliable marker during acute changes in renal function [122]. Firstly, using SCr to assess true kidney function has several limitations [123]. A significant decline in GFR can be observed before it is reflected in an increase SCr (up to 50% of renal function could be lost before SCr might change). Secondly, SCr does not reflect renal function during acute changes until a steady state have been reached, which may last for several days. Moreover, SCr is a poor indicator for AKI due principally because it could not help to diagnose early AKI and differentiate the various causes of AKI. Concerning equations estimating GFR, within the 5^th^ and 95^th^ percentile for age, both MDRD and Cockcroft-Gault formulas provide comparable data consistent with values obtained for age-specific historic inulin clearance [124]. The Cockcroft-Gault formula gave higher values at younger ages, and lower values in the older than 70 years than the obtained with the MDRD equation [125]. The CKD-EPI formula was formed to provide a more precise estimate of GFR among individuals with normal or only slightly declined GFR (i.e, >60 mL/min per 1.73 m^2^) [126]. Currently, CKD-EPI formula is recommended to assess kidney function, screening and diagnosis of CKD according to KDIGO guidelines [80]. However, this formula was not validated in cancer patients. It should also be stressed that people older than 65 years usually have, but not always lowered GFR [127, 128]. It may be explained by the presence of other comorbidities, which may influence GFR [129]. This is of particular interest in cancer patients. Drug dosing guidelines have historically been developed using the Cockcroft–Gault formula to assess kidney function. Most pharmacokinetic studies for drug dosing in renal disease were performed using the Cockcroft–Gault formula because it was recommended by the FDA (Food and Drug Administration) prior to publication of the MDRD study equation [130].

#### Kidney function and risk of death in cancer

Possible association between renal function and death in malignancy are not widely studied [131–137]. In some reports, inverse associations with different cutoff value of the eGFR and mortality were observed [131–134], whereas in others there were no relationships [135, 136]. In the recent study, Lichtman et al. [138] reported that after controlling for standard prognostic factors, renal function was not predictive of either overall survival or recurrence-free survival in subjects>65 years with early breast cancer regardless of regimen. It worth to stress that cancer itself may have a strong effect on mortality rather than an impaired renal function. Some studies suggested that the association of eGFR and mortality depend upon the type of cancer [131, 139]. These inconsistencies may be due to the sample size in the various eGFR levels, threshold value of eGFR, formula used to assess eGFR, cancer stage, and follow-up periods, different population in regard to the treatment (treatment naive or treated patients). In the recent retrospective study on 9465 subjects with newly diagnosed malignancy, Yang et al. [88] reported that presence of a eGFR below 60 mL/min/1.73 m2 or proteinuria were associated with higher risk for all-cause death. In addition, subgroup analysis revealed that eGFR<60 mL/min/1.73m2 was an independent predictor of mortality in patients with hematologic and gynecological malignancies, but not in those with other types of malignancy. In addition, proteinuria appeared as a risk for death among patients with digestive system cancer. It should be also pointed out that Yang et al. [88] enrolled incident rather than prevalent patients and eGFR by CKD-EPI, not MDRD was obtained at the time of diagnosis, thus they could rule out the impact of anticancer treatment on renal function. The authors also stressed that the prevalence of CKD was frequent (32,4%) in patients with new diagnosis of malignancy. Proteinuria, being a risk factor for the progression of CKD should be assessed and monitored in cancer patients. This is crucial due to the potential effect of proteinuria on survival. As the data are limited and inconsistent, it appears that the mechanisms underlying the impact of renal function on mortality among patients with different primary cancer are complex and remain to be elucidated in large and prospective studies.

#### Therapeutic implications of CKD presence in cancer patients

It has been reported that incidence of CKD is high in cancer patients. It is essential to stress that SCr is not appropriate for assessment of kidney function. GFR is estimated by equations such as MDRD, also in subjects with a normal SCr and even on regular visits in subjects coming for routine check-ups without administration of antineoplastic medications. It should be also considered that oncology patients are still exposed to renal and extrarenal toxicity of non-antineoplastic medications prescribed for other reasons. Janus et al. [82] found that in 46.7% of the 120 subjects with a GFR below 60 ml/min/1.72m^2^ and for which drug dosages were available in the medical file were administered at least one medication with an unadjusted dose in relation to kidney function. It is very important for clinicians to handle antineoplastic drugs properly in this population. Approximately 50% of all chemotherapeutics are excreted predominantly by the kidneys in urine as unchanged drug or active metabolite(s), thus, any impairment in kidney function may lead to accumulation of potentially toxic metabolites and overdosage [78]. The dose of anticancer drugs in CKD patients should be adjusted to avoid severe toxicities [140]. In addition, using chemotherapeutics with potential nephrotoxicity will also require specific monitoring and, when available, specific prevention reducing the risk for nephrotoxicity, especially in patients with preexisting CKD [78]. It was shown very elegantly in the IRMA-1 study, where 79.9% patients were given at least one drug needed a dose adjustment or for which there were no data available for use in patients impaired kidney function [78]. In addition, 80.1% of the patients were administered at least one nephrotoxic medication [78]. In the BIRMA study, 24.8% patients were ‘chemotherapy-naïve' and 75.2% patients were not “chemotherapy-naïve' i.e. at least one antineoplastic agent was administered [82]. The prevalence of CKD in ‘not chemotherapy-naïve' was a significantly higher than in ‘chemotherapy-naïve' patients. In the BIRMA study, 54.3% of the ‘chemotherapy-naïve' subjects exhibited a GFR below 90mL/min/1.73m^2^ relative to 67.1% in ‘not chemotherapy-naïve' subjects (P<0.0001). Three IV bisphosphonates were prescribed in BIRMA study i.e. ibandronic, pamidronic, and zoledronic acid. The majority of subjects with bone metastases received zoledronic acid (220 patients). It is of interest that 67.3% had an impaired GFR and 50.9% of them were classified as having CKD [82]. In stage 2 CKD subjects, potential nephrotoxicity of the therapy is the important and relevant issue. It has been shown that preexisting impairment in kidney function is a risk factor for nephrotoxicity caused by anticancer treatment [141]. Thus, in patients with worsened kidney function, clinicians should take into account the potential risk of nephrotoxicity, and implement preventive measures whenever possible. However, in a case when administration of nephrotoxic agent is necessary, it is essential to adjust the dose, according to the kidney function and to follow the guidelines for the management of nephrotoxicity if available, as in a case of cisplatin [139]. In the BIRMA study, subjects with impaired kidney function were administered a mean of 1.2 nephrotoxic antineoplastic agent [82]. In addition, some patients were given nephrotoxic combination, exposing them to an elevated iatrogenic nephrotoxicity (i.e gemcitabine + cisplatine). Thus, it is essential to avoid (whenever possible), nephrotoxic associations of antineoplastic and other agents. In the BIRMA study, there are no data available on other potentially nephrotoxic drugs, i.e. pain killers, therefore it was not possible to assess the number of potentially nephrotoxic drugs (antineoplastic and others) administered in this study [82]. Therefore, they considered that the exposure to nephrotoxic agents was underestimated in the population studied. It is plausible that nephrotoxicity induced by antineoplastic agents, i.e. zoledronic acid, contribute to the increased prevalence of CKD, but other factors should be also taken into account. The prevalence of a decreased GFR (<90mL/min/1.73m^2^) was significantly increased in BIRMA relative to IRMA trial (64.0 vs 52.9%, P<0.0001) [50] and when compared to the study of Dogan et al. [76]. It may be due the fact that different patient populations were studied. In BIRMA study, breast cancer patients constituted a high proportion, whereas Dogan et al. [76] included mainly patients with gastrointestinal cancers. In addition, age was also significantly different in these three studies. It is of utmost importance to be aware of the kidney function in subjects administered with nephrotoxic or potentially nephrotoxic agent, and to monitor kidney function regularly, before each course of chemotherapy. This problem was addressed recently in the CALGB (Cancer and Leukemia Group B) 49907 study assessing the effect of preexisting kidney function and five end-points: toxicity, dose modification, therapy completion, relapse-free survival and overall survival in patients aged more than 65 years old with early breast cancer on routine treatment, i.e. cyclophosphamide / doxorubicin (AC) or cyclophosphamide / methotrexate / fluorouracil-CMF over capecitabine. Lichtman et al. [138] reported that incidence of stage 3 or 4 CKD was high reaching up to 72% for CMF, 64% for AC and 75% in capecitabine group. They also observed that baseline kidney function estimated using Cockcroft-Gault formula was highly related to the occurrence of nonhematologic toxicity for the AC regimen and very mildly for the capecitabine regimen, but not related for the CMF regimen [138]. There currently are no standard recommendations for cancer screening in the dialysis population (ie, NKF-KDOQI [National Kidney Foundation−Kidney Disease Outcomes Quality Initiative] or KDIGO guidelines). In practice, screening for malignancy in dialysis subjects has been given with an individualized patient-focused approach on the basis of the patient's cancer risk factors, expected survival, and transplantation status [112]. CANcer and DialYsis (CANDY) is the retrospective multicenter study, on 178 chronic dialysis patients with malignancy. Majority of the patients in this study, received at least one chemotherapeutic agent requiring either dose adjustments (72%) or adequate time of administration (82%) [142]. Iatrogenic toxicity developed in 44% of the treated patients: 34% was related to agents needed dose adjustment, and 17% was attributed to the additional agents with no existing therapy recommendations in dialyzed population [142]. The authors stressed that evidence is lacking in regard to use of systemic chemotherapy in CKD including RRT and this led to the inappropriate use of chemotherapeutic drugs and lethal toxicity in this particular population. Boesler et al. [143] demonstrated that administration of chemotherapeutic agents is feasible in hemodialyzed patients without unpredictable severe unwanted effects. They reported that the dosages given were significantly higher relative to proposed adjustments according to Dettli’s proportional dose reduction rule [144]. They administered additionally the dose calculated by Dettli’s rule to supplement the agent cleared by hemodialysis. They concluded that chemotherapy was feasible, but selection of the appropriate dose still needed the decision of medical oncology and nephropharmacology specialists, as there were no algorithms and no evidence available.

### Kidney transplantation and cancer

#### Kidney recipients with a history of cancer

Kidney allograft transplantation is the best available option of treatment for most patients with ESRD [145, 146]. As subjects with CKD often have comorbidities, including past malignancy [147–150], the assessment of potential renal allograft recipient should be efficient and cost effective. Generally, most clinical guidelines suggest a waiting period free of recurrence of two to five years for most patients with a history of carcinoma [151–156]. However, the questions on the sufficient time after treatment of the malignancy and waitlisting for kidney transplantation are still the matter of debate. It comes from the assumption that immunosuppression therapy may enhance development of micrometastasis [157] thus increase the risk of recurrence. Data on tumor recurrence after transplantation is scarce and tumor type is associated a marked variability in the likelihood of recurrence [158]. It determines the recommendations for cancer survivors taking into consideration patient and tumor characteristics [159, 160]. In general, patients with history of basal or squamous skin cancer, *in situ* bladder cancer, all noninvasive papillary tumors of the bladder, and asymptomatic solitary renal cell cancers <5 cm can be waitlisted without delay [150, 152, 160]. In a case of malignant melanoma, colorectal carcinoma other than *in situ* Duke's A or B1 carcinoma, invasive cervical cancer, breast cancer with regional node involvement, bilateral disease, or inflammatory histology five years without evidence of recurrence is required [150, 152, 160]. Patients with ductal carcinoma *in situ* may be waitlisted after two years’ interval. The low recurrence rates (below 10%) were reported for localized renal cell carcinoma (RCC); testicular, cervical, and thyroid cancers; and lymphomas (including Hodgkin and non-Hodgkin lymphoma, higher recurrence rates (between 10 and 25%) were noted for uterus, colon, prostate, and breast cancer and Wilms tumor, while the highest rates (over 25%) were recorded for bladder carcinoma, advanced renal cell carcinoma, sarcomas, myelomas, and both melanoma and nonmelanoma skin cancers [159, 160].

#### Oncological therapy in kidney allograft recipients

Solid organ transplantation is associated with higher incidence of malignancy development relative to the general population [161] and several, but not all, studies have demonstrated increased cancer-related mortality among transplant recipients [162–164]. This excessive death rate in organ transplant recipients may be due to previous malignancy as well as to the fact that immunosuppressive therapy may promote more aggressive cancer development due to the loss of immune surveillance and/or due to the concern of organ rejection [153, 165]. Thus, patients are offered less aggressive anticancer treatment [153, 165]. Controversies existing around cancer screening in kidney transplant recipients in regard to reduced life expectancy and competing causes of death were presented elegantly by Acuna et al. [166] in systematic review of clinical practice guidelines. Oncological management in kidney transplant recipients is challenging and results from the balance between treatment of the malignancy and maintenance of a sufficient graft function. Recently, Wanchoo et al. [167] discussed the use of immune checkpoint inhibitors (ICI) in kidney transplant recipients. They summarized the 8 published cases when ICI were used in kidney transplant patients. They stressed that the transplant community should take into account the potential risk of rejection in renal allograft recipients treated with ICI. They also presented a novel strategy to prevent rejection in transplant recipients receiving PD-1 inhibitors using pre-emptive steroids and sirolimus. However, there is not enough data to give specific recommendations for oncology treatment in kidney transplant recipients. Each case should be considered individually and decision should be based on the patient’s priority after receiving consultation from oncologist and transplant physician. The potential for graft loss needs to be weighed against the natural history and stage of the malignancy. The reasonable approach is to diminish immunosuppression, and consider switch into a mammalian target of rapamycin inhibitor [168]. In some case discontinuation of immunosuppression may be appropriate.

## SUMMARY

Increased incidence of CKD, in particular, in the elderly, are of utmost importance. Many antineoplastic agents are cleared primarily by the kidneys as unchanged drugs or active metabolites. Therefore, a decline in kidney function can potentially lead to alterations in pharmacokinetics, elevated blood levels of the drugs, and increased toxicity. It has been shown that a remarkable number of CKD subjects treated with chemotherapy require dose reduction in case of CKD, but they are not administered the adjusted dose [82]. Thus, it should be stressed that CKD is underrecognized problem in oncology population and eGFR is to be assessed simultaneously, not only in oncology ward but also in every department. This is due to the fact that patients are getting older, have more comorbidities, are administered more potentially nephrotoxic drugs and undergone more potentially nephrotoxic procedures such as percutaneous coronary interventions-PCI or CT with IV contrast agent etc. [169]. It is of utmost importance to be aware of the kidney function in patients receiving nephrotoxic or potentially nephrotoxic agent and to monitor kidney function regularly, before each course of chemotherapy. Oncologists should adjust the dose of cytotoxic drugs according to actual kidney function. Besides, in patients treated with nephrotoxic chemotherapeutic agents in particular with preexisting impairment of kidney function, the necessity of concomitant drugs should be carefully evaluated i.e.NSAIDS. They should be avoided, if possible, as they may contribute to the nephrotoxicity of chemotherapeutics.

## Abbreviations

AC: cyclophosphamide/doxorubicine
AKI: acute kidney injury
ALK: anaplastic lymphoma kinase)
BIRMA: Belgian Renal Insufficiency and Anticancer Medications
BRAF: v-Raf murine sarcoma viral oncogene homolog B)CALGB-Cancer and Leukemia Group B
CI: confidence interval
CIN: contrast-induced nephropathy
CHF: chronic heart failure
CKD: chronic kidney disease
CKD-EPI: chronic kidney disease- epidemiological collaboration
CMF: cyclophosphamide/ methotrexate/ fluorouracil
CT: computed tomography
CTLA-4: cytotoxic T-lymphocyte antigen 4
DM: diabetes mellitus
DTPA: diethylene-triamine-pentaacetate
ECOG: Eastern Cooperative Oncology Group
EDTA: ethylenediaminetetraacetic acid
ESRD: end-stage renal disease
ESUR: European Society of Urological Radiology
eGFR: estimated glomerular filtration rate
EGFR: epidermal growth factor receptor)
FDA: Food and Drug Administration
FSGS: focal segmental glomerulosclerosis
HER2: human epidermal growth factor receptor-2
HR: hazard ratio
ICI: immune checkpoint inhibitors
ICU: intensive care unit
IRMA: Insuffisance Rénale et Médicaments Anticancéreux; Renal Insufficiency and Anticancer Medications
IV: intravenous
KDIGO: Kidney Disease: Improving Global Outcomes
MDRD: Modification of Diet in Renal Disease
mTOR: mammalian target of rapamycin
NKF-KDOQI: National Kidney Foundation−Kidney Disease Outcomes Quality Initiative
NSAIDs: non-steroidal anti-inflammatory drugs
OR: odds ratio
PCI: percutaneous coronary interventions
PD-1: programmed cell death protein 1
PDL: 1igand of programmed cell death protein 1
RANKL: (receptor activator of nuclear factor kappa beta ligand)
PSA: prostate-specific antigen
RCC: renal cell carcinoma
RIFLE: risk, injury, failure, loss of function, end-stage kidney disease)
RRT: renal replacement therapy
TKI: tyrosine kinase inhibitor
TMA: thrombotic microangiopathy
VEGR: vascular endothelial growth factor
VEGFR: vascular endothelial growth factor receptor
